# A nearly complete database on the records and ecology of the rarest boreal tiger moth from 1840s to 2020

**DOI:** 10.1038/s41597-022-01230-8

**Published:** 2022-03-25

**Authors:** Ivan N. Bolotov, Mikhail Yu. Gofarov, Evgeny S. Koshkin, Vyacheslav V. Gorbach, Yury I. Bakhaev, Oleg E. Berlov, Sergey Yu. Gordeev, Yulia S. Kolosova, Alexander V. Kondakov, Alexey V. Korshunov, Grigory S. Potapov, Sergey Yu. Sinev, Spiridon S. Sleptsov, Vitaly M. Spitsyn, Evgeny G. Strelnikov, Andrey V. Timchenko, Risto Haverinen, Kari Nupponen, Hannu Saarenmaa

**Affiliations:** 1 Northern Arctic Federal University, Northern Dvina Emb. 17, 163002 Arkhangelsk, Russia; 2 N. Laverov Federal Center for Integrated Arctic Research of the Ural Branch of the Russian Academy of Sciences, Northern Dvina Emb. 23, 163000 Arkhangelsk, Russia; 3 Institute of Water and Ecology Problems of the Far Eastern Branch of the Russian Academy of Sciences, 56 Dikopoltsev Str., 680000 Khabarovsk, Russia; 4 Petrozavodsk State University, Lenin Av. 33, 185910 Petrozavodsk, Russia; 5 Lipetsk, Russia; 6 Irkutsk Anti-Plague Research Institute of Siberia and the Russian Far East, Trilisser Str. 78, 664047 Irkutsk, Russia; 7 Institute of General and Experimental Biology of the Siberian Branch of the Russian Academy of Sciences, Sakhyanova Str. 6, 670047 Ulan-Ude, Russia; 8 Institute of Human Ecology of the Siberian Branch of the Russian Academy of Sciences, Leningradski Av. 10, 650065 Kemerovo, Russia; 9 Zoological Institute of the Russian Academy of Sciences, Universitetskaya Emb. 1, 199034 Saint-Petersburg, Russia; 10 Yakut Science Centre of Complex Medical Problems of the Siberian Branch of the Russian Academy of Sciences, Yaroslavskogo Str. 6/3, 677000 Yakutsk, Russia; 11 Yugansky State Nature Reserve, Ugut Village, 628458 Surgut District, Khanty-Mansi Region, Russia; 12 Moscow, Russia; 13 Vantaa, Finland; 14 Espoo, Finland; 15 Department of Forest Sciences, University of Helsinki, PO Box 27, 00014 Helsinki, Finland

**Keywords:** Phenology, Population dynamics, Entomology

## Abstract

Global environmental changes may cause dramatic insect declines but over century-long time series of certain species’ records are rarely available for scientific research. The Menetries’ Tiger Moth (*Arctia menetriesii*) appears to be the most enigmatic example among boreal insects. Although it occurs throughout the entire Eurasian taiga biome, it is so rare that less than 100 specimens were recorded since its original description in 1846. Here, we present the database, which contains nearly all available information on the species’ records collected from 1840s to 2020. The data on *A. menetriesii* records (*N* = 78) through geographic regions, environments, and different timeframes are compiled and unified. The database may serve as the basis for a wide array of future research such as the distribution modeling and predictions of range shifts under climate changes. It represents a unique example of a more than century-long dataset of distributional, ecological, and phenological data designed for an exceptionally rare but widespread boreal insect, which primarily occurs in hard-to-reach, uninhabited areas of Eurasia.

## Abstract (summary)

**Table:** 

Measurement(s)	specimen record • biological parameters of specimen • environmental characteristics • sampling date • habitat image • specimen image
Technology Type(s)	digital curation
Factor Type(s)	year • month • ten-day period • day • geographic location • altitude • habitat • landscape type • presence of waterbody • ecoregion • developmental stage • sex • individual condition
Sample Characteristic - Organism	Menetries’ Tiger Moth *Arctia menetriesii* (Insecta: Lepidoptera: Erebidae)
Sample Characteristic - Environment	taiga biome • high-altitude environment
Sample Characteristic - Location	Northern Eurasia

## Background & Summary

Recent climate changes and habitat loss significantly influence the distribution and abundance of invertebrates at different spatial scales, from local and regional to the global^1–5^. The order Lepidoptera (butterflies and moths) is among most imperiled insect groups worldwide^6–9^, sharing high rates of decline and even extinction^10–13^. The tiger moths are a monophyletic clade (tribe Arctiini) belonging to the family Erebidae^14–17^. This group contains a number of large and colorful species, many of which are rare and endangered^14^. Tropical areas of Asia, Australasia, Africa, and South America represent a hotspot of tiger moths’ species richness globally^18–25^, while a few species are distributed in boreal forest and Arctic tundra biomes^26–31^.

**Fig. 1:**
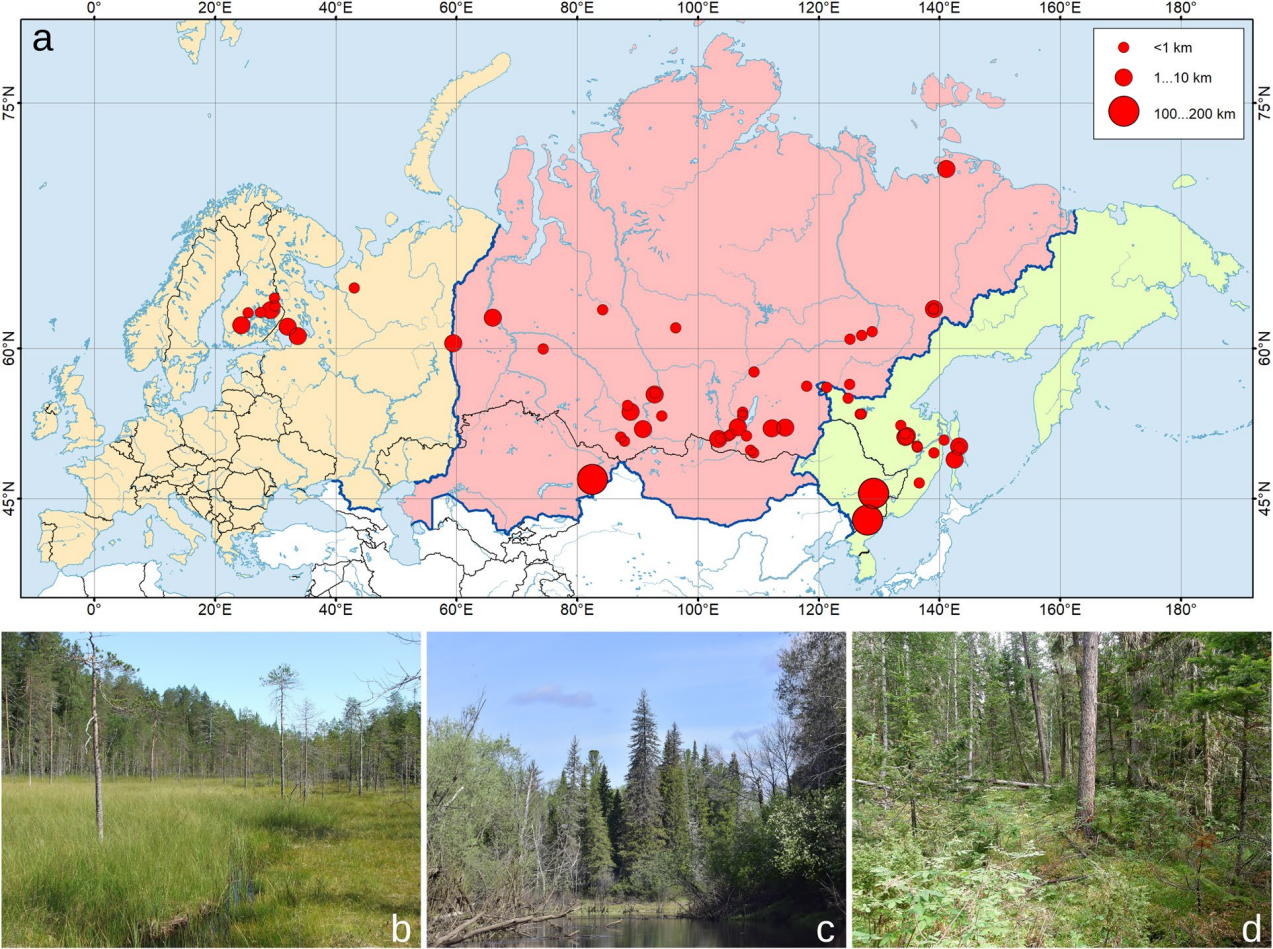
Localities and habitats of *Arctia menetriesii*. (**a**) Map of records listed in the database^52^. The size of circles indicates the uncertainty of the geographic co-ordinates (see Legend). The color areas represent three regions discussed in this study as follows: *yellow* Europe (records from Finland, Northern European Russia, and the Urals); *red* Siberia (records from Western and Eastern Siberia in Russia, and East Kazakhstan); and *green* the Far East (records from the Russian Far East and northeastern China). The map was created using ESRI ArcGIS 10 software (https://www.esri.com/arcgis); the topographic base of the map was created with Natural Earth Free Vector and Raster Map Data (https://www.naturalearthdata.com) and Global Self-consistent Hierarchical High-resolution Geography, GSHHG v. 2.3.7 (https://www.soest.hawaii.edu/wessel/gshhg). (Map: Mikhail Y. Gofarov). (**b**–**d**) Examples of habitat images linked to the database: (**b**) half-open bog surrounded by primeval coniferous forest, Kuhmo, Finland (Photo: Risto Haverinen); (**c**) primeval coniferous taiga forest in the Negusyakh River valley, Yugansky State Nature Reserve, Western Siberia, Russia (Photo: Evgeny G. Strelnikov); (**d**) primeval coniferous taiga forest near the Bolshoy Anay River, Baikalo-Lensky Nature Reserve, Eastern Siberia, Russia (Photo: Oleg E. Berlov).

**Fig. 2:**
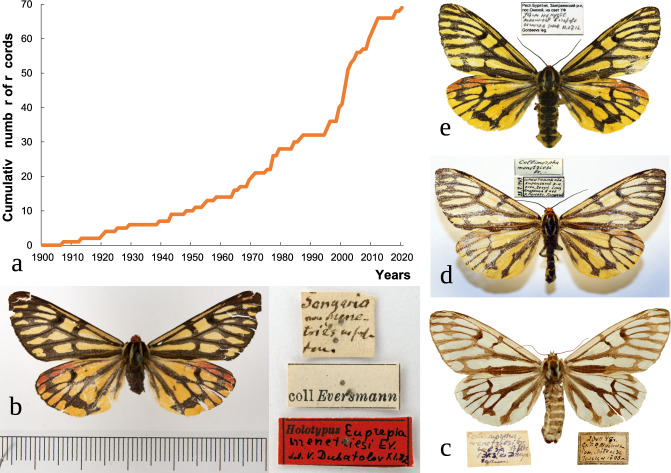
Number of records and examples of specimen images of *Arctia menetriesii*. (**a**) Cumulative number of records throughout the entire species’ range over the past 120 years (1900–2020)^52^. (**b**-**e**) Examples of imago specimen images linked to the database:^52^ Image AM-075_SP-01_SYS: “Songoria” [=East Kazakhstan], before 1846, holotype ♂ [coll. ZIN] (Photo: Sergey Y. Sinev) (**b**); Image AM-071_SP-01_ESK: the top of Arsenyeva Mt., Sikhote-Alin Mts, Far East, Russia, 20.vii.1948, ♀, Kononov & Kurentzov leg. [coll. FSCEATB FEB RAS] (Photo: Evgeny S. Koshkin) (**c**); Image AM-028_SP-01_OEB: Chechui River near the mouth of Rassokha River, Irkutsk Oblast, Siberia, Russia, 22.vii.1969, ♀, Pleshanov leg. [coll. of E. & O. Berlov, Irkutsk, Russia] (Photo: Oleg E. Berlov) (**d**); Image AM-035_SP-04_SYG: Onokhoy, Uda River valley, Republic of Buryatia, Siberia, Russia, 11.vii.2012, ♀, Gordeev leg. [coll. IGEB] (Photo: Sergey Y. Gordeev) (**e**).

The Menetries’ Tiger Moth *Arctia menetriesii* (Eversmann, 1846) may be considered the rarest and most enigmatic species among boreal representatives of the tribe Arctiini^32,33^. This species has a continuous range expanding throughout the entire Eurasian taiga biome from Finland to the Sakhalin Island and northeastern China^26,32,34^ (Fig. 1a). Despite its enormous range, Menetries’ Tiger Moth occurs very rarely, and less than 100 specimens were recorded since its initial description in 1846^32,35,36^ (Fig. 2a–e). It could primarily be found in hard-to-reach, uninhabited areas of the continent, harboring primeval woodlands^32^ (Fig. 1b–d). Earlier, this species was placed within its own monotypic genus, *Borearctia* Dubatolov, 1984^33^ but it was recently transferred to the genus *Arctia* Schranck, 1802 based on a comprehensive multi-locus phylogenetic research^14^. Host plants and life history of *A. menetriesii* were discussed in a few works^36–39^ and are rather poorly known.

The holotype specimen of this species (Fig. 2c,d) is imprecisely labelled as being collected from a broad historical region, the Songoria (currently eastern Kazakhstan; most likely somewhere within the Tarbagatai or Saur Mountains)^33,35,40,41^. Next records come from Finland^37,42,43^, Yakutia^44^, North Manchuria^34^, and Sakhalin^34^ in the 1910s-1920s. Kurentzov^44^ presented the first range map of *A. menetriesii* with eight records known to date (1965). Later, several authors listed additional records from the Urals and Asiatic Russia^28,32,33,39,45–48^. The species was considered extinct in Finland^49^ but was rediscovered in 2003^50,51^. Dubatolov^26^ published a more detailed range map with 40 records (37 precise points and three vague localities) but, unfortunately, any data on certain records has not been listed. Our team started to compile information of *A. menetriesii* records in the early 1990s. The first georeferenced dataset was published in 2013^32^. This compilation contained the data on 43 records, including three samples from vague localities such as Lake Baikal^32^.

**Fig. 3:**
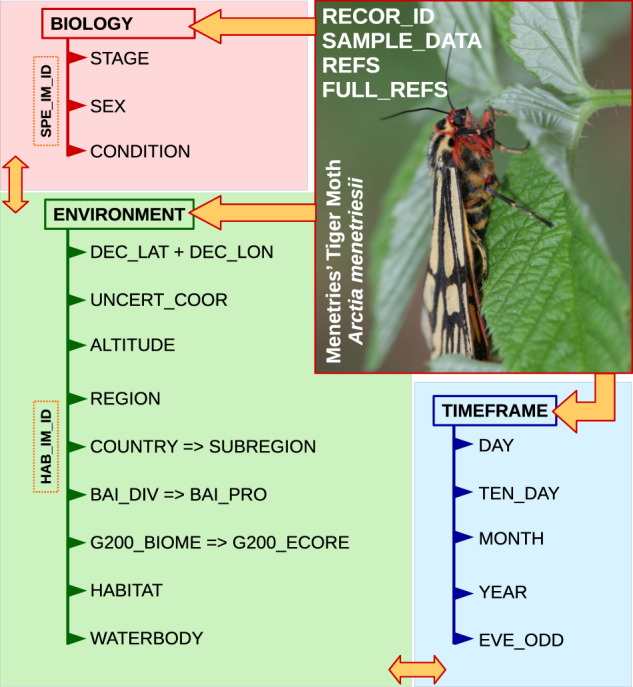
Structure of the Menetries’ Tiger Moth Range and Ecology Database (1840s-2020)^52^. The field names are decoded in Online-only Table 1. The blocks containing environmental, temporal, and biological categories are green, blue, and red, respectively. The linked data (images) is highlighted by dashed orange frame. Living female moth: image AM-035_SP-01_SYG.jpeg linked to the database, Onokhoy settlement, Uda River valley, Republic of Buryatia, Russia. (Graphics: Ivan N. Bolotov; Photo: Sergey Y. Gordeev).

**Fig. 4:**
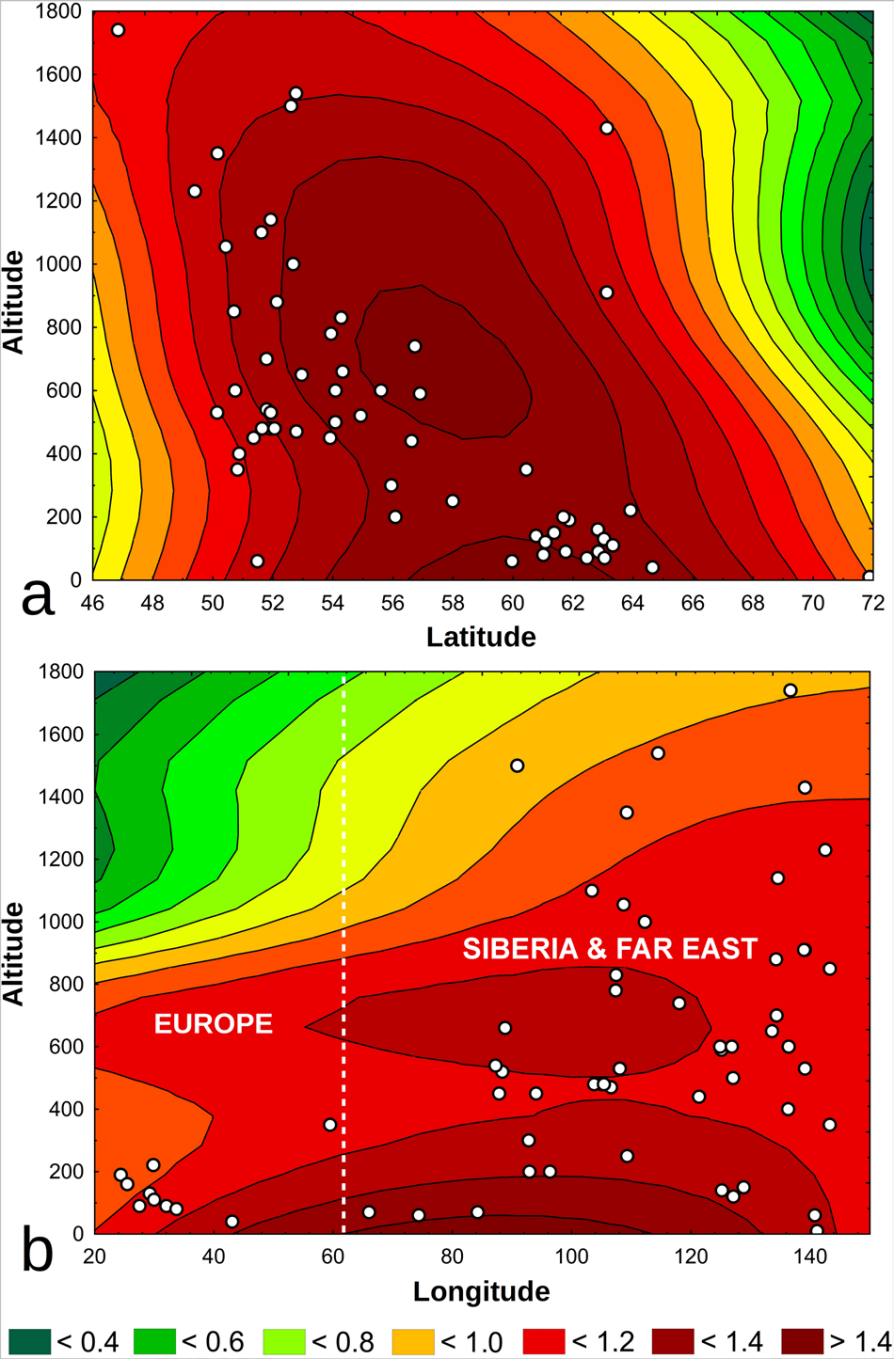
3D contour plots of the number of collected individuals of *Arctia menetriesii* against geographic variables (altitude and geographic co-ordinates) of the localities based on the Menetries’ Tiger Moth Range and Ecology Database (1840s-2020):^52^ (**a**) number of collected individuals vs latitude and altitude; and (**b**) number of collected individuals vs longitude and altitude. The white circles indicate the species’ localities. The color areas and corresponding legend show the spline interpolation of the number of collected individuals per locality. In particular, the yellow to green areas indicate the lack of records, the orange to red areas indicate the prevalence of singleton records, and the darker red areas indicate the possibility to find more than one specimen per locality under these conditions. The white dashed line separates approximate areas corresponding to Europe and Asia. The 3D plots were created using v. Statistica 13.3 (StatSoft®, TIBCO Software Inc., CA, USA).

**Fig. 5:**
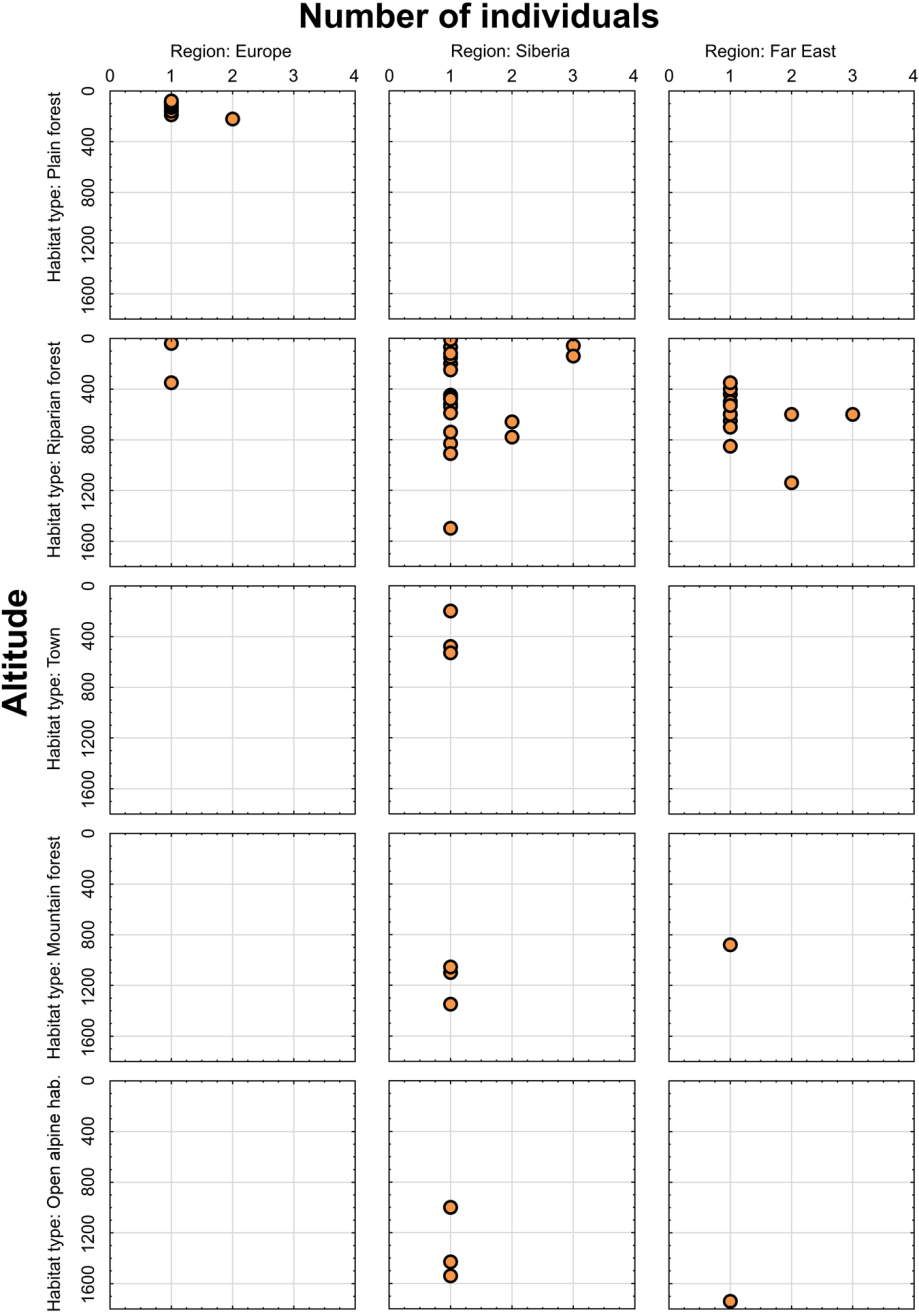
Scatterplot showing number of collected individuals of *Arctia menetriesii* plotted against altitude of the collecting locality and categorized by habitat type and region. The scatterplot was created using v. Statistica 13.3 (StatSoft®, TIBCO Software Inc., CA, USA) based on the Menetries’ Tiger Moth Range and Ecology Database (1840s-2020)^52^.

**Fig. 6:**
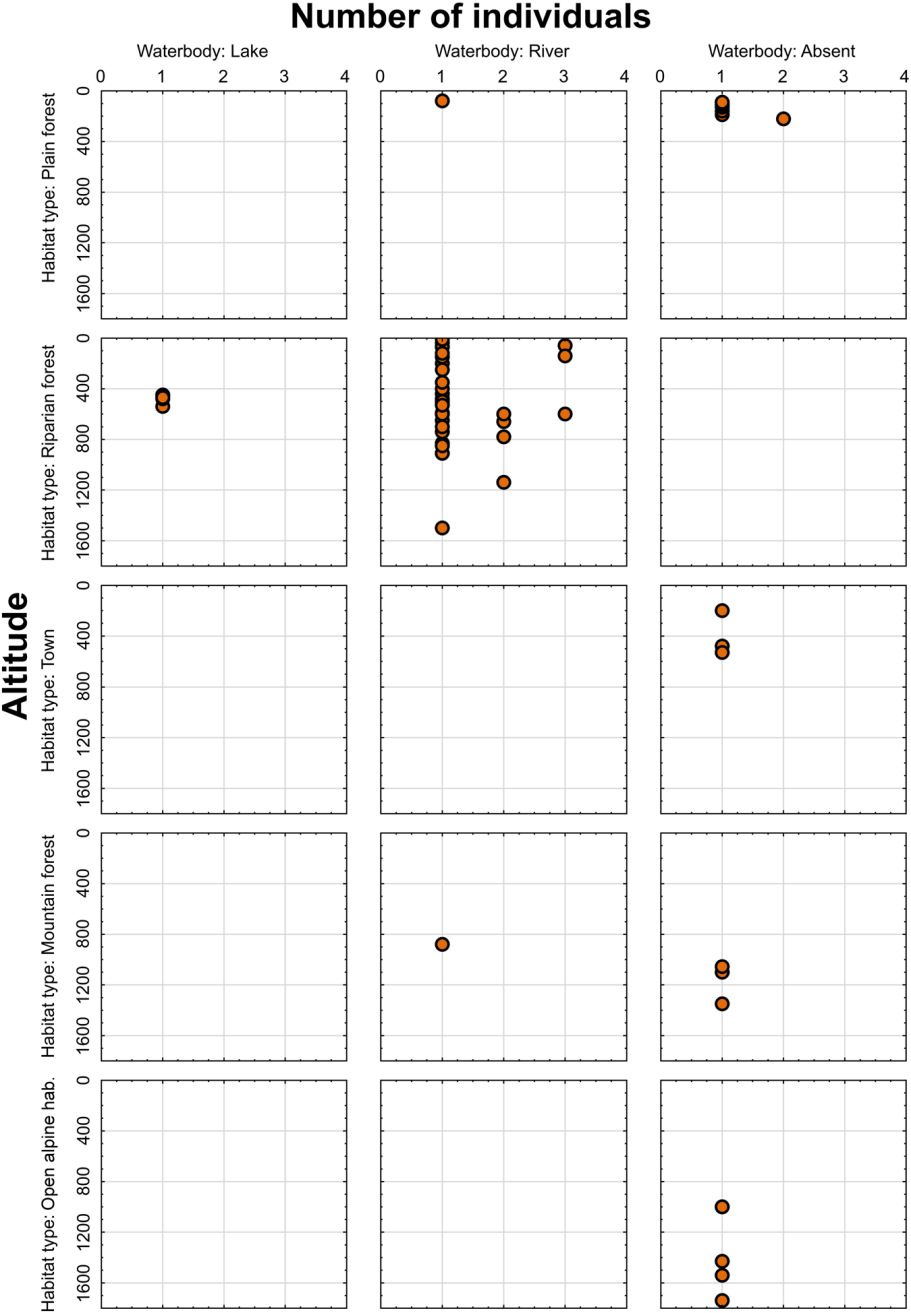
Scatterplot showing number of collected individuals of *Arctia menetriesii* plotted against altitude of the collecting locality and categorized by habitat type and the presence of waterbody at the locality. The scatterplot was created using Statistica v. 13.3 (StatSoft®, TIBCO Software Inc., CA, USA) based on the Menetries’ Tiger Moth Range and Ecology Database (1840s-2020)^52^.

The present study describes our final compilation, the Menetries’ Tiger Moth Range and Ecology Database (1840s-2020)^52^, which contains nearly all available information on this extremely rare species collected since its original description (Fig. 3). In total, the database comprises geographic, environmental, and temporal information on 78 records of *A. menetriesii* (Online-only Table 1, Figs. 4–6). Here, we consider individual specimens as separate records with unique IDs, even if these specimens were collected simultaneously from the same locality. All the records were delineated into three larger geographic regions: Europe (Finland, Northern European Russia, and the Urals), Siberia (Western and Eastern Siberia in Russia, and East Kazakhstan), and the Far East (Russian Far East and northeastern China) (Fig. 1a). Available images of habitats and samples are linked to the database via file names (examples: Figs. 1b–d and 2b–e). The database is a unique example of a more than century-long dataset of distributional, ecological, and phenological data designed for a rare boreal moth, having a broad trans-Eurasian range.

The present work was also motivated by a broader aim linked to historical and recent rapid defaunation processes on Earth^53,54^ and, more precisely, to the so-called insect apocalypse in the Anthropocene^2,3,5^. A plethora of recent surveys revealed that there is a drastic decline in insect (and moths in particular) abundance and biomass over the past few decades^11,55,56^. The causal mechanisms and drivers of this phenomenon are poorly understood^57–59^ but, at first glance, could be linked to agricultural intensification, pesticide use, urbanization, habitat loss, and climate warming^3,60,61^. Conversely, it was shown that many insect species show no declines and that the most dramatic examples of declines come from highly populated agricultural areas of the Northern Hemisphere^10,62^.

Based on these considerations, *A. menetriesii* may be used as a model organism to address the long-term distribution and abundance shifts in larger boreal insects, having a broad trans-Eurasian range. Hence, the Menetries’ Tiger Moth Range and Ecology Database (1840s-2020)^52^ could serve as the reliable basis for a variety of future research such as distributional and phenological modeling and predictions of range shifts under various climate change scenarios.

## Methods

### Original field research

The authors of this paper were looking for *A. menetriesii* specimens during their fieldworks starting from the early 1990s (ca. 30-year period). The fieldworks covered Finland (R.H., K.N., H.S.), Northern European Russia and the Urals (I.N.B., M.Y.G., Y.S.K, G.S.P, V.M.S., H.S.), Western Siberia (E.G.S.), Eastern Siberia (O.E.B., S.Y.G., S.S.S., H.S.), and the Russian Far East (E.S.K., Y.I.B.). The samples were collected by hands or by an entomological net. Here, we present eight unpublished records collected by ourselves. Additionally, a few own records were published in our earlier works^28,32,36,38,39^.

### Collection lots and published data

First, data on collected specimens of Menetries’ Tiger Moth were obtained from a wide array of museum and private collections in Finland, Germany, Lithuania, and Russia. Specimens of *A. menetriesii* are available in not less than 12 museum collections as follows: Museum Witt [**MWM**], München, Germany; Finnish Museum of Natural History [**MZH**], Zoology Unit, Helsinki, Finland; Institute of General and Experimental Biology of the Siberian Branch of the Russian Academy of Sciences [**IGEB**], Ulan-Ude, Russia; Russian Museum of Biodiversity Hotspots [**RMBH**], Federal Center for Integrated Arctic Research, the Ural Branch of the Russian Academy of Sciences, Arkhangelsk, Russia; Siberian Institute of Plant Physiology and Biochemistry of the Siberian Branch of the Russian Academy of Sciences [**SIFIBR**], Irkutsk, Russia; Sakhalin Regional Museum [**SARM**], Yuzhno-Sakhalinsk, Russia; Siberian Zoological Museum [**SZMN**], Institute of Animal Systematics and Ecology of the Siberian Branch of the Russian Academy of Sciences, Novosibirsk, Russia; Tobolsk Regional Museum [**TORM**], Tobolsk, Russia; Zoological Institute of the Russian Academy of Sciences [**ZIN**], Saint Petersburg, Russia; Zoological Museum of the Moscow University [**ZMMU**], Moscow, Russia; Federal Scientific Center of the East Asia Terrestrial Biodiversity of the Far East Branch of the Russian Academy of Sciences [**FSCEATB FEB RAS**], Vladivostok, Russia; and Institute for Biological Problems of Crylitozone of the Siberian Branch of the Russian Academy of Sciences [**IBPC**], Yakutsk, Russia. Additional samples of this rare species are kept in at least 10 private collections as follows: Finland Research Collection of R. Haverinen, Helsinki, Finland; private collection of T. & K. Nupponen, Espoo, Finland; private collections of H. Saarenmaa, M. Tähtinen, J. Tiittanen, K. Silvonen & R. Leinonen, Helsinki, Finland; private collection of V. Višinskas, Vilnius, Lithuania; private collection of A. Korshunov, Kemerovo, Russia; private collection of E. & O. Berlov, Irkutsk, Russia; private collection of Y. Sidelnikov, Khabarovsk, Russia; private collection of E. Koshkin, Khabarovsk, Russia; private collection of A. Timchenko, Moscow, Russia; and private collection of Yasunori Kishida, Tokyo, Japan. Here, we present 10 unpublished collection records, as well as corrected or expanded data on five published records.

Second, we searched for published records of *A. menetriesii* through online tools such as Web of Science, Scopus, and Google Scholar using the scientific name of this species (in combination with both generic names: *Borearctia* and *Arctia*) and its English, Finnish, and Russian common names (“Menetries’ Tiger Moth”, “idänsiilikäs”, and “медведица Менетрие”, respectively) as keywords. Additional data was collected from references cited in the works we have found. None of the public records was found in the iNaturalist online portal (https://www.inaturalist.org).

We consider that the Menetries’ Tiger Moth Range and Ecology Database (1840s-2020)^52^ represents a nearly complete resource on available records of *A. menetriesii*. Perhaps, a few additional specimens may be found in museum and private collections in China (not checked by us), and some singletons in private collections of Russian and European amateurs may have been overlooked. However, we estimate that the amount of records overlooked by us (if any) may not be higher than 8–12 (10–15% of the total number of records in our database) due to an exceptional rarity of this species.

### Data processing

The collecting localities were georeferenced and verified using the Google Earth tool (https://www.google.com/intl/ru/earth). The same tool was used to assess the altitude of each locality, which was rounded to the nearest tenth. The altitude of mountain localities was additionally checked using available topographic maps and the ACE2 global digital elevation model^63^. More than half of the localities were precisely georeferenced (±10…500 m; *N* = 50; 64.1% of the total number of records). Other part of the localities share less precise co-ordinates (±1…10 km; *N* = 22; 28.2% of the total number of records). Four records (AM-075, AM-076, AM-077, and AM-078) were ascribed for vague localities^34,35^, and their co-ordinates listed in the database are approximate (±100…200 km). Finally, two records (AM-036 and AM-053) were left without co-ordinates because their localities were too vague for referencing purposes (the entire Lake Baikal and an unknown mountain in Yakutia, respectively).

Unfortunately, the holotype specimen of *A. menetriesii* (AM-075) is also among the group of rather uncertain records. The label of the holotype reads as “Songoria” (Fig. 2b). The original description states that “Haec *Eupreria*, ab illustrissimo D. Ménétriés ad describendum mihi communicata, campos Songariae inhabitat”^35^. In this case, Songoria most likely means the so-called Songoria rossica [=Russian Dzhungaria; currently East Kazakhstan]. This region contains desert areas and the Tarbagatai and Saur mountain ranges. Based on available data on the habitat spectrum of this species, it became clear that the holotype was collected somewhere in the mountains.

The Tarbagatai Mountains as the most probable type locality of the species first appeared in the Palearctic Lepidoptera catalogue of Staudinger and Rebel in 1901 but with a question mark^40^. Subsequent researchers in Finland and Russia followed the catalogue, placing the type locality within that mountain range^37,41,42,64^. Later, Dubatolov assumed that the type locality might be somewhere within the Saur Range^33^, which represents the eastern extremity of the Tarbagatai. In a subsequent work, Dubatolov listed the type locality as follows: “Mountains around Lake Zaysan, East Kazakhstan Region, Kazakhstan”^65^. This large lake is situated between the Southern Altai and Tarbagatai mountains. As a conclusion, the type locality of *A. menetriesii* cannot be identified with certainty but it is most likely somewhere within the Tarbagatai – Saur mountain system in East Kazakhstan. We therefore placed it in the middle of this range but with a high uncertainty level (±200 km).

Each georeferenced locality was assigned to the corresponding Bailey’s Ecoregion Division and Province^66^ using ESRI ArcGIS 10 software (https://www.esri.com/arcgis). The Bailey’s Ecoregion maps were downloaded from the UN Environment World Conservation Monitoring Centre (UNEP-WCMC), Cambridge, United Kingdom (https://www.unep-wcmc.org/resources-and-data/baileys-ecoregions-of-the-world). Ecoregion divisions are coded as follows: tundra (*TD*); Subarctic (*SD*); Subarctic regime mountains (*SM*); prairie (*PD*); prairie regime mountains (*PM*); warm continental regime mountains (*WM*); and hot continental regime mountains (*HM*). Ecoregion provinces are numbered as follows: (1) Arctic tundra; (2) continental and extreme continental light deciduous taiga; (3) continental dark evergreen needle-leaf taiga; (4) eastern oceanic taiga; (5) moderate continental dark evergreen needle-leaf taiga; (6) forest-creeping trees-tundra of extreme continental climate; (7) forest-tundra of moderately continental and continental climate; (8) open woodland-creeping trees-tundra; (9) open woodland-tundra; (10) continental steppe-forest-tundra and steppe-forest meadows; (11) oceanic forest-creeping trees; (12) forest-alpine meadows; and (13) small-leafed and coniferous wooded steppes of continental climate.

The WWF’s Global 200 Project established a system of Earth’s ecoregions and biomes^67,68^, which is widely used in biogeographic, ecological, and conservation surveys^69,70^. We therefore placed each georeferenced locality within these categories using ESRI ArcGIS 10 software (https://www.esri.com/arcgis).

All types of habitat were converged into five larger categories: mountain forest; plain forest; riparian forest (forest patches in river valley, stream valley or on lake shore); alpine meadows and tundra (open alpine habitats); and town (urban environments). For example, two specimens (AM-006 and AM-007) were collected in a half-open bog site surrounded by coniferous forest in Kuhmo, Finland. This habitat was assigned to the plain forest category. In each case, the original information on habitat and available data on plant cover at the collecting site could be seen in the “SAMPLE_DATA” field^52^.

The Julian calendar date of a historical sample (AM-043) collected in Russia before 1918 was changed to the Gregorian calendar (actual) date but both the historical and the actual date could be seen in the “SAMPLE_DATA” field^52^.

## Data Records

The Menetries’ Tiger Moth Range and Ecology Database (1840s-2020) can be downloaded from *figshare*^52^. The main database is presented in XLSX format (ArcMenDB_1840_2020.xlsx). The corresponding reference list and museum collection codes are available as separate PDF files (ArcMenDB_RefList.pdf and ArcMenDB_Museum_ID.pdf, respectively). These files and the ZIP-archive with images (ArcMenDB_Images.zip) can be downloaded together with the database.

Each field name of the database is decoded and explained in Online-only Table 1. The database structure is illustrated in Fig. 3. A unique code (RECOR_ID) is assigned for each record listed in the database. Available label and field observation data on each sample of *A. menetriesii* is presented in the “SAMPLE_DATA” column (as a brief description). The sources of information for a given specimen are cited in the “REFS” (=References) column, while the full references for each record are listed in the “FULL_REFS” column. The complete list of references can also be downloaded as a separate PDF file (see above).

Information in the database is clustered into three large blocks: “Environment”, “Timeframe”, and “Biology” (Fig. 3). The “Environment” block contains geographic and environmental data on collecting localities such as the co-ordinates, their uncertainty, altitude, region, country, ecoregions (Bailey’s and WWF Global 200), habitat type, and the presence/absence of a waterbody. Available habitat images (format: JPEG and TIFF) are linked to this block via file names. The “Timeframe” block presents information on the day, ten-day-period, month, and year of a given record, as well as in which (odd or even) year it was collected. Finally, the “Biology” block contains information on a given specimen as follows: developmental stage (larva, imago, etc.), sex (imaginal records only), and condition (living or died). Available specimen images (both living and collection specimens; format: JPEG and TIFF) are linked to this block via file names.

Each parameter, factor levels, and linked data used in the database are described in Online-only Table 1. Furthermore, in this table we list the number of records, for which this parameter/factor/linked data is available. In total, the database contains 78 records but some of them lack certain data points as follows: co-ordinates (2), altitude (6), habitat (7), presence/absence of a waterbody (8), Bailey’s Ecoregion (2), The Global 200 Biome/Ecoregion (2), collecting day (17), ten-day period (14), month (11), year (7), sex (20), and condition (3). The habitat and specimen images are available for 10 and 29 records, respectively.

The geographic coverage of the database can be seen in Fig. 4a,b. The color areas on this figure indicate the approximate level of probability to find *A. menetriesii* specimens under certain geographic conditions based on the spline interpolation of the number of collected individuals per locality. The set of available georeferenced localities expands from 42.4°N to 71.9°N by latitude, from 24.4°E to 143.3°E by longitude, and from 10 to 1740 m by altitude. From the majority of the localities, singleton specimens were collected. However, there are eight localities with two or three recorded specimens in each, indicating the presence of permanent populations in these sites (one in Europe and seven in Siberia and the Far East; Fig. 4b). The environmental coverage of the database in relation to the altitude of collecting localities and the number of collected individuals is visualized in Figs. 5–6. Most non-singleton samples were collected from lowland to upland riparian forests of Siberia and the Far East between 0 and 1,200 m altitude (Fig. 5). Furthermore, the vast majority of specimens, including almost all non-singleton records, was sampled near a river or stream (Fig. 6).

## Technical Validation

Experienced entomologists verified all the records and ecological data included to the database. Furthermore, we used only records that were based on collected specimens. None of the doubtful records such as those based solely on visual observations has been included. We cited and list corresponding references for records obtained from reliable published sources, while the museum storage was listed for every collection sample, if available. An uncertainty of geographic co-ordinates for each locality was estimated. Finally, all the authors carefully checked the complete database for possible technical failures and errors.

## Data Availability

No code was used in this study.

## Online-only Table

**Online-only Table 1:** Description of each parameter and factor levels used in the Menetries’ Tiger Moth Range and Ecology Database (1840s-2020).

Field name	Parameters, factors, and linked data	Data type	Data group	Brief description	Number of records, for which this parameter/factor/linked data is available
RECOR_ID	Record ID	Text	ID	The unique ID that was assigned to each available specimen record (AM-001 – AM-078)	78
SAMPLE_DATA	Locality and sample data	Text	Primary survey data	Brief description of available label and field observation data for each specimen such as the locality, habitat, date, developmental stage, sex, condition, behavior, and name of collector(s). The current depository abbreviation, if available, is presented as well	78
REGION	Region name	Text	Environment	The records were separated into three large regions as follows: Europe (with the Urals); Siberia; and the Far East	78
COUNTRY	Country name	Text	Environment	The species records are situated in four countries: China, Finland, Kazakhstan, and Russia	78
SUBREGION	Subregion name	Text	Environment	As a subregion, we list administrative divisions of the corresponding countries such as provinces and regions	78
DEC_LAT	N (latitude)	Decimal	Environment	Latitudinal co-ordinate of the collecting locality	76
DEC_LON	E (longitude)	Decimal	Environment	Longitudinal co-ordinate of the collecting locality	76
UNCERT_COOR	Co-ordinate uncertainty (m)	Integer	Environment	It ranges from 10 to 200,000 m	76
ALTITUDE	Altitude (m)	Integer	Environment	Altitude of the sampling locality. It was estimated based on the co-ordinates with lower uncertainty (≤10,000 m) only	72
HABITAT	Habitat	Text	Environment	Five factor levels were applied as follows: mountain forest; alpine meadows and tundra; plain forest; riparian forest; and town	71
WATERBODY	Presence of waterbody at the collection site	Text	Environment	Three factor levels were used as follows: absent; lake shore; and river (=stream) valley	70
BAI_DIV	Bailey’s Ecoregion Division	Text	Environment	Abbreviation of the corresponding division, which was estimated based on the co-ordinates. The abbreviations of divisions are given in the Methods section	76
BAI_PRO	Bailey’s Ecoregion Province	Integer	Environment	Numerical code of the corresponding province, which was estimated based on the co-ordinates. The codes of provinces are given in the Methods section	76
G200_BIOME	The Global 200 Biome	Text	Environment	Name of the corresponding WWF’s Global 200 biome, which was estimated based on the co-ordinates	76
G200_ECORE	The Global 200 Ecoregion	Text	Environment	Name of the corresponding WWF’s Global 200 ecoregion, which was estimated based on the co-ordinates	76
DAY	Day	Integer	Timeframe	Day, on which the specimen was collected	61
TEN_DAY	Ten-day period	Integer	Timeframe	Ten-day period, on which the specimen was collected	64
MONTH	Month	Text	Timeframe	Month, in which the specimen was collected	67
YEAR	Year	Integer	Timeframe	Year, in which the specimen was collected (three specimens were listed with uncertain year as follows: 1840s; before 1926; and before 1982)	74
EVE_ODD	Even or odd year	Text	Timeframe	Two factor levels were used as follows: even vs odd	71
STAGE	Stage	Text	Biology	Two factor levels were used as follows: larva vs adult (imago)	78
SEX	Sex	Text	Biology	Two factor levels were used as follows: male vs female (for imaginal samples only)	58
CONDITION	Condition	Text	Biology	Two factor levels were used as follows: living vs dead	75
HAB_IM_ID	Habitat image ID	Text	Linked data (image)	The unique file ID corresponding to available images of a given habitat. This file ID was created using the record ID + HB (habitat) + number of image + name of photographer [abbreviation] + file format, for example: AM-035_HB-01_SYG.jpeg. The number of available image files is given in square brackets	10
SPE_IM_ID	Specimen image ID	Text	Linked data (image)	The unique file ID corresponding to available images of a given specimen. This file ID was created using the record ID + SP [specimen] + number of image + name of photographer [abbreviation] + file format, for example: AM-035_SP-01_SYG.jpeg. The number of available image files is given in square brackets	29
REFS	References	Text	Sources of information	Citation of information sources for each record as follows: Author(s) (Year)	78
FULL_REFS	Full references	Text	Sources of information	Full reference(s) to each record. The complete list of references is also deposited with the database as a supplementary PDF file.	78
